# Response to Matters Arising about the etiology of ventilator-associated pneumonia in the European POS-VAP cohort

**DOI:** 10.1186/s13054-026-06138-y

**Published:** 2026-06-18

**Authors:** Marlieke E. A. de Kraker, Holly Jackson, Ana Catalina Hernandez Padilla, C. Henri van Werkhoven, Bruno Francois

**Affiliations:** 1 Infection Control Program, Faculty of Medicine, Geneva University Hospitals, World Health Organization Collaborating Center, Geneva, Switzerland; 2 Saw Swee Hock School of Public Health, ADVANCE-ID Network, National University of Singapore, Singapore, Singapore; 3 Department of Respiratory and Infectious Diseases, Lee Kong Chian School of Medicine, Nanyang Technological University, Singapore, Singapore; 4 Inserm CIC 1435, CHU Limoges, Limoges, France; 5 Inserm UMR 1092, Université de Limoges, Limoges, France; 6 Service de Réanimation Polyvalente, CHU Limoges, Limoges, France; 7 Julius Center for Health Sciences and Primary Care, University Medical Center Utrecht, Utrecht University, Utrecht, The Netherlands

Dear Editor,

We would like to thank Ignacio Martin-Loeches and Luis Felipe Reyes [1] for their correspondence concerning our study on incidence, aetiology and outcomes of ventilator-associated pneumonia (VAP) in Europe [2].

It is well-known that micro-organisms responsible for VAP vary by geographic region, patient case mix, duration of hospital stay before onset, and risk factors for colonisation/infection with multi-drug resistant pathogens [3, 4]. Thus, it is not surprising that several large cohort studies describing VAP aetiology rank causative pathogens differently. Generally, most VAP are caused by Gram-negative pathogens, including *Pseudomonas aeruginosa*, *Klebsiella pneumoniae*, or *Enterobacter* or *Acinetobacter* species, but an important proportion is caused by Gram-positive pathogens, among which *Staphylococcus aureus* is the most dominant species [5]. In our cohort, Gram-negative pathogens are also implicated in the majority (237/359, 66%) of microbiologically evaluable VAP cases. Nevertheless, at the species level, *S. aureus* (26.2%) was the most common pathogen (3.9% methicillin resistant *S. aureus* [MRSA], 17.5% methicillin susceptible *S. aureus* [MSSA], 4.7% without data), followed by *Haemophilus influenzae* (16.2%), and *P. aeruginosa* (15.0%) [2]. Despite Martin-Loeches and Reyes [1] highlighting the importance of *P. aeruginosa* VAP in the ENIRRI cohort, *S. aureus* VAP is also frequent in this patient population; combining the reported MRSA (21/556, 3.8%) and MSSA VAP (56/556, 10.1%) gives an overall proportion of 13.9% (95%CI 11.0–16.6), only slightly lower than *P. aeruginosa* VAP (101/556, 18.9%, 95%CI 15.0-21.3) [6]. Similarly, in the TAVem cohort, 24% of VAP cases could be attributed to *P. aeruginosa* as well as *S. aureus* (MRSA 2%, MSSA 22%) [7]. While reported *S. aureus* VAP proportions vary slightly, all cohorts confirm the increasing importance of MSSA over MRSA, aligned with previous findings in VAP [4] and bloodstream infections in Europe [8].

The higher proportion of *P. aeruginosa* VAP in TAVem and ENIRRI, could be related to country selection; both include countries from South-America, where the epidemiology is expected to be different from Europe. While country-specific VAP aetiology is not available for these cohorts, an ENIRRI sub-analysis showed that Argentina and Germany report a high proportion of *P. aeruginosa* nosocomial respiratory tract infections [9], neither are included within POS-VAP. In line with our country-level results, the EU VAP/CAP study [10], including healthcare-associated pneumonia and VAP, shows that *S. aureus* was the dominant strain in Spain, France, and Belgium, while *P. aeruginosa* was most common in Italy. *Acinetobacter* species were the most frequently identified VAP pathogens in Croatia, Romania and Serbia [2]. This highlights the importance of reporting VAP epidemiology by country.

Patient case mix is another important consideration when interpreting reported VAP incidence and aetiology. POS-VAP [2], ENIRRI [6], and TAVem [7] included broadly similar populations, with VAP detected predominantly in males (study proportions: 71–72%), around 60 years of age (study median age: 58–62), with a high proportion of comorbidities, like diabetes (17–19%), and severity scores indicating a high predicted mortality risk (40–46%). Reasons for admission, however, differed. While POS-VAP predominantly included mixed ICUs, 29.5% of ENIRRI VAP cases were identified in medical ICUs [6], where patients are more likely to have prior healthcare exposure. In POS-VAP, important admission diagnoses among VAP cases included stroke (12.4%), trauma (11.2%), and traumatic brain injury (9.2%) [2], conditions associated with an increased risk of *S. aureus* and *H. influenzae* VAP [4, 11, 12]. Consistent with this (Table 1), our data shows that *S. aureus* is the highest ranked VAP pathogen in patients admitted with traumatic brain injury (10/24, 41.7%), stroke (15/49, 30.6%) and trauma (14/48, 29.2%), and it was most prevalent in patients with cardiac decompensation (5/7, 71.4%) or acute myocardial infarction (4/8, 50%). In contrast, *P. aeruginosa* predominated for more rare diagnoses (‘other primary diagnosis’ 11/38, 28.9%), cardiovascular surgery (4/14, 28.6%), and sepsis/septic shock (4/18, 22.2%). In ENIRRI [6], 26.6% of VAP patients had septic shock on admission, compared with 8% in POS-VAP [2]. Together with the higher proportion of medical ICU patients, this may partly explain the higher proportion of *P. aeruginosa* VAP in ENIRRI.

**Table 1:**
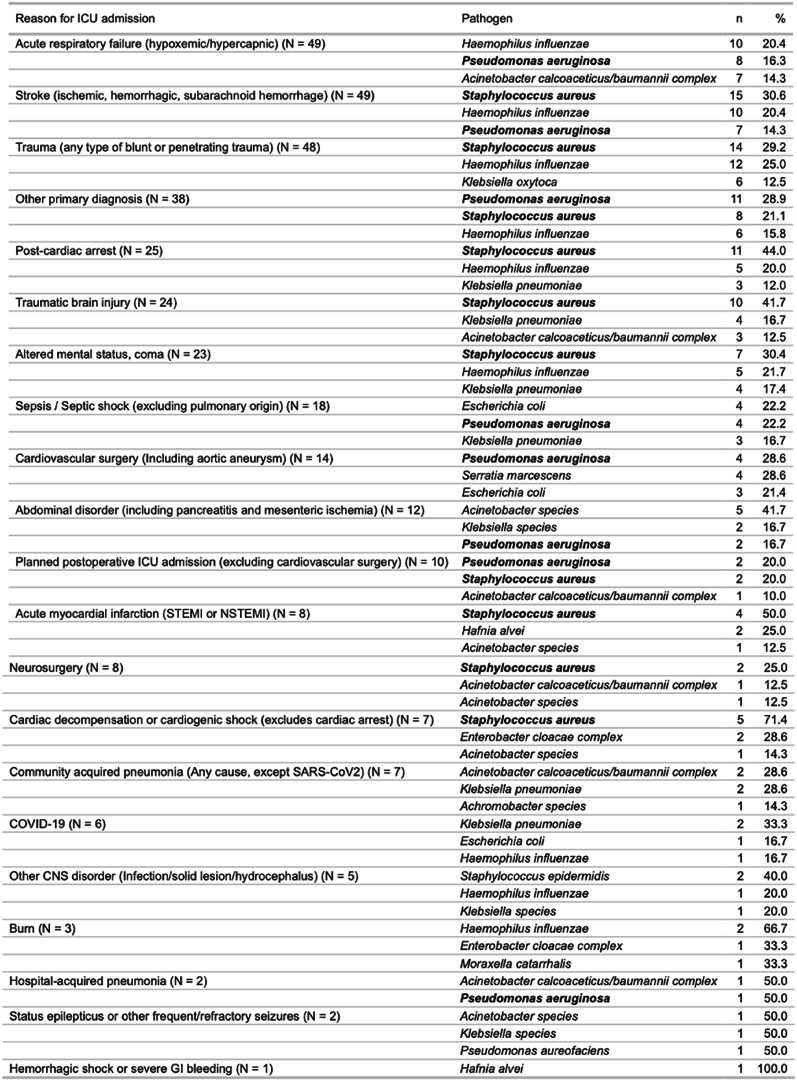
Top three pathogens associated with ventilator-associated pneumonia, stratified by admission diagnosis (N=number of microbiologically evaluable [MBE] patients with this diagnosis), among patients in the POS-VAP cohort with microbiological documentation (MBE N=359, n=512 unique pathogen-patient combinations)

A difference in pathogen distribution has also been described for early (≤ 4 days) versus late VAP [13]. As previously reported [2], *S. aureus* was the most common pathogen in both groups (28.5% and 25.0%, respectively), but the proportion of *P. aeruginosa* increased from 8.1% to 18.6%, comparing early versus late VAP. As POS-VAP only included first VAP episodes during the first period of invasive mechanical ventilation (IMV), a larger proportion of early VAP cases (31.4%) may have been recorded compared to other VAP cohorts, increasing the importance of *S. aureus* VAP. Unfortunately, the proportion of early and late VAP is rarely reported in other studies, and it is often unclear how repeated episodes per patient are accounted for. Our stringent criteria avoided double counting of multiple VAP episodes or recurrences in the same patient. As such, POS-VAP data should be considered reflective of VAP occurrence in ICU patients during their first IMV episode.

There is no uniform definition for VAP diagnosis, but most definitions rely on a chest image suggestive of pneumonia combined with the presence of one or more clinical signs and symptoms in patients under IMV for at least 48 h [14]. In addition, microbiological testing can be performed, using blood and/or (invasive) respiratory samples [3]. Within POS-VAP, active VAP screening was encouraged based on FDA criteria, while microbiological confirmation reflected routine clinical practice and was available for 60.7% of VAP cases [2] compared to 75% within ENIRRI [6]. This difference can partly be explained by our stringent 2-day time window around VAP diagnosis date, to ensure identified organisms were associated with the recorded infection. Discrepancies between clinically- and cohort-defined VAP may have also reduced the frequency of microbiological testing, although discordance was low at 5.1%. Microbiological confirmation in POS-VAP was based predominantly on respiratory samples (81.6% endotracheal aspirate [ETA], 15.0% bronchoalveolar lavage [BAL], 5.8% blood culture). In contrast, blood cultures were the main source of pathogen identification within ENIRRI (blood 81.3%, BAL 63.3%, ETA/Sputum 44.8%) [6] and TAVem (blood 74.5%, BAL 28.2%, ETA 68.6%) [7]. Greater reliance on blood cultures likely reflects inclusion of patients with bacteraemic or late-onset VAP, more frequently involving *P. aeruginosa*, while predominance of ETA samples in POS-VAP may reflect earlier, less severe VAP episodes, although increased detection of respiratory colonisation cannot be ruled out.

High-level VAP surveillance data from multinational cohorts, like POS-VAP, TAVem, or ENIRRI, should not be interpreted as direct guidance for empirical prescribing at the individual patient-level. Hospitals should utilise their own data to inform prescribing guidelines, ideally stratifying microbiological data by ward and infection type to ensure treatment strategies reflect the true ecological context [15]. Therefore, POS-VAP provides feedback to participating sites about their local data and encourages data re-use. In settings where the number of ICU patients, or specifically VAP cases, is low, or routine reporting is limited, national or international surveys can provide supportive information, whereby detailed, stratified results can help determine local applicability.

Prospective cohort data are especially useful for benchmarking across settings and over time, highlighting shifts in incidence, aetiology, or antimicrobial resistance. Differences in VAP incidence, despite similar patient case mix, may indicate gaps in infection prevention and control or antibiotic stewardship strategies. In our cohort [2], adherence to VAP prevention bundle elements ranged from 59.1% (daily sedation vacation) to 93.1% (head-of-bed elevation) and varied between ICUs. Further evaluation of individual bundle elements and their association with VAP risk could help optimise prevention strategies.

To conclude, there is large heterogeneity in VAP aetiology across countries, ICUs, and patient case mix, which complicates the direct comparison of findings between cohorts. Nevertheless, they provide useful evidence to inform pathogen prioritisation for research and development. In addition, data from the POS-VAP network can support site selection and facilitate the design of randomised controlled trials evaluating pathogen-specific preventive or therapeutic interventions in VAP. Based on the findings across studies, it is clear that *S. aureus* and *P. aeruginosa* are key targets for improved VAP intervention strategies to enhance clinical outcomes for critically ill patients in Europe.

## Data Availability

The data analysed within is from the prospective, observational POS-VAP study and are not publicly available. However, data access for secondary analyses can be requested from the POS-VAP scientific coordinators (BF, MdK, and CHvW ).

## References

[1] Martin-Loeches I, Reyes LF. Letter to perpetual observational study of the clinical and microbiological epidemiology of ventilator-associated pneumonia in Europe. Crit Care. 2026;30(1):167. 41957639 10.1186/s13054-026-06007-8 PMC13067484

[2] Jackson H, Hernandez Padilla AC, Vintcent LEM, Barac A, Cremer O, Daix T, et al. Perpetual observational study of the clinical and microbiological epidemiology of ventilator-associated pneumonia in Europe. Crit Care. 2026;30(1):112. 41821089 10.1186/s13054-025-05753-5 PMC12980874

[3] Torres A, Niederman MS, Chastre J, Ewig S, Fernandez-Vandellos P, Hanberger H, et al. International ERS/ESICM/ESCMID/ALAT guidelines for the management of hospital-acquired pneumonia and ventilator-associated pneumonia: guidelines for the management of hospital-acquired pneumonia (HAP)/ventilator-associated pneumonia (VAP) of the European Respiratory Society (ERS), European Society of Intensive Care Medicine (ESICM), European Society of Clinical Microbiology and Infectious Diseases (ESCMID) and Asociación Latinoamericana del Tórax (ALAT). Eur Respir J. 2017;50(3):170058. 10.1183/13993003.00582-2017 28890434

[4] Hurley JC. World-wide variation in incidence of *Staphylococcus aureus* associated ventilator-associated pneumonia: a meta-regression. Microorganisms. 2018;6(1):18. 29495472 10.3390/microorganisms6010018 PMC5874632

[5] Sader HS, Streit JM, Carvalhaes CG, Huband MD, Shortridge D, Mendes RE, et al. Frequency of occurrence and antimicrobial susceptibility of bacteria isolated from respiratory samples of patients hospitalized with pneumonia in Western Europe, Eastern Europe and the USA: results from the SENTRY antimicrobial surveillance program (2016–19). JAC-Antimicrobial Resist. 2021;3(3):dlab117. 10.1093/jacamr/dlab117 PMC8522161 34671728

[6] Martin-Loeches I, Reyes LF, Nseir S, Ranzani O, Povoa P, Diaz E, et al. European Network for ICU-Related Respiratory Infections (ENIRRIs): a multinational, prospective, cohort study of nosocomial LRTI. Intensive Care Med. 2023;49(10):1212–22. 37812242 10.1007/s00134-023-07210-9 PMC10562498

[7] Martin-Loeches I, Povoa P, Rodríguez A, Curcio D, Suarez D, Mira JP, et al. Incidence and prognosis of ventilator-associated tracheobronchitis (TAVeM): a multicentre, prospective, observational study. The Lancet Respiratory Medicine. 2015;3(11):859–68. 26472037 10.1016/S2213-2600(15)00326-4

[8] European Centre for Disease Prevention and Control (ECDC). Antimicrobial resistance in the EU/EEA (EARS-Net) - Annual Epidemiological Report for 2024. Stockholm: ECDC; 2025.

[9] Serrano-Mayorga CC, Olivella-Gomez J, Sanabria-Herrera N, Nseir S, Torres A, Martin-Loeches I, et al. Pseudomonas aeruginosa in Patients With Nosocomial Respiratory Infections: A Secondary Analysis of the European Network for ICU-Related Respiratory Infections. Chest. 2026;169(5):1240–54. 41520818 10.1016/j.chest.2025.12.033

[10] Koulenti D, Tsigou E, Rello J. Nosocomial pneumonia in 27 ICUs in Europe: perspectives from the EU-VAP/CAP study. Eur J Clin Microbiol Infect Dis. 2017;36(11):1999–2006. 27287765 10.1007/s10096-016-2703-z

[11] Robba C, Rebora P, Banzato E, Wiegers EJA, Stocchetti N, Menon DK, Citerio G. Collaborative European Neuro Trauma Effectiveness Research in Traumatic Brain Injury Participants and Investigators. Incidence, Risk Factors, and Effects on Outcome of Ventilator-Associated Pneumonia in Patients With Traumatic Brain Injury: Analysis of a Large, Multicenter, Prospective, Observational Longitudinal Study. Chest. 2020;158(6):2292–303. 32634435 10.1016/j.chest.2020.06.064

[12] Kasuya Y, et al. Ventilator-associated pneumonia in critically ill stroke patients: frequency, risk factors, and outcomes. J Crit Care. 2011;26(3):273–9. 10.1016/j.jcrc.2010.09.006 10.1016/j.jcrc.2010.09.006 21106334

[13] Kalanuria AA, Zai W, Mirski M. Ventilator-associated pneumonia in the ICU. Crit Care. 2014;18(2):208. 25029020 10.1186/cc13775 PMC4056625

[14] Fally M, Haseeb F, Kouta A, Hansel J, Robey RC, Williams T, et al. Unravelling the complexity of ventilator-associated pneumonia: a systematic methodological literature review of diagnostic criteria and definitions used in clinical research. Crit Care. 2024;28(1):214. 38956655 10.1186/s13054-024-04991-3 PMC11221085

[15] Kalil AC, Metersky ML, Klompas M, Muscedere J, Sweeney DA, Palmer LB, et al. Management of Adults With Hospital-acquired and Ventilator-associated Pneumonia: 2016 Clinical Practice Guidelines by the Infectious Diseases Society of America and the American Thoracic Society. Clin Infect Dis. 2016;63(5):e61–111. 27418577 10.1093/cid/ciw353 PMC4981759

